# Machine Learning–Driven Risk Prediction Model in Transthyretin Amyloid Cardiomyopathy

**DOI:** 10.1001/jamacardio.2026.3496

**Published:** 2026-08-28

**Authors:** Giovanni Baj, Nicola Ciocca, Pooya Mohammadi Kazaj, Xuan Ma, Annina A. Studer Bruengger, Simon F. Stämpfli, Niklas F. Ehl, Sarah Hugelshofer, Otmar Pfister, Joëlle Lehmann, Christoph Ryffel, Lukas Hunziker, Michael Poledniczek, Andreas Kammerlander, George C. M. Siontis, Stephan Windecker, Moritz J. Hundertmark, Christian Nitsche, Isaac Shiri, Christoph Gräni

**Affiliations:** 1 Department of Cardiology, Inselspital, Bern University Hospital, University of Bern, Bern, Switzerland; 2 Department of Digital Medicine, University of Bern, Bern, Switzerland; 3 Graduate School for Cellular and Biomedical Sciences, University of Bern, Bern, Switzerland; 4 Department of Cardiology and Institute for Radiology and Nuclear Medicine, Stadtspital Triemli, Zurich, Switzerland; 5 Heart Center Lucerne, Luzerner Kantonsspital, Lucerne, Switzerland; 6 Center for Molecular Cardiology, University of Zurich, Zurich, Switzerland; 7 Department of Cardiology, HOCH, Cantonal Hospital St Gallen, St Gallen, Switzerland; 8 Cardiology Unit, Cardio-Vascular Department, University Hospital of Canton Vaud, Centre Hospitalier Universitaire Vaudois, Lausanne, Switzerland; 9 Department of Cardiology, University Heart Center, University Hospital Basel, University of Basel, Basel, Switzerland; 10 Division of Cardiology, Department of Internal Medicine II, Medical University of Vienna, Vienna, Austria; 11 Associate Editor, *JAMA Cardiology*

## Abstract

**Importance:**

Transthyretin cardiac amyloidosis (ATTR-CM) is associated with poor prognosis and significant morbidity and mortality, yet existing staging systems have limited ability to accurately predict outcomes in contemporary patient populations.

**Objective:**

To develop and validate a machine learning (ML)–based prognostic model for patients with ATTR-CM.

**Design, Setting, and Participants:**

This multicenter cohort study, including specialized referral centers for cardiac amyloidosis, used data from patients with confirmed ATTR-CM in the Swiss Cardiac Amyloidosis Registry (February 2018 to November 2025) and the Cardiac Amyloidosis Registry of the Medical University of Vienna (July 2014 to February 2025). A random survival forest model was developed and evaluated through internal-external cross-validation, with each center iteratively held out for validation. The model was compared with the National Amyloidosis Centre and Mayo Clinic staging systems. Data analysis was conducted from December 2025 to February 2026.

**Exposure:**

Clinical, demographic, medication, laboratory, and echocardiographic variables were used to train an ML-based time-to-event prediction model.

**Main Outcomes and Measures:**

The primary outcome was defined as a composite event of all-cause mortality and hospitalization for heart failure.

**Results:**

A total of 850 patients (median [IQR] age, 79 [74-83] years; 750 [88.2%] male) were included across 3 cohorts (from Bern, Switzerland [Bern-Swiss cohort], n = 352; the other centers from Switzerland combined [Other-Swiss cohort], n = 260; and Vienna, Austria [Vienna cohort], n = 238). The random survival forest model demonstrated good discrimination across all held-out cohorts, with Harrell concordance indices of 0.74 (95% CI, 0.69-0.78), 0.77 (95% CI, 0.69-0.83), and 0.72 (95% CI, 0.67-0.77) for the Bern-Swiss, Other-Swiss, and Vienna cohorts, respectively, and 3-year area under the curve (AUC) of 0.73 (95% CI, 0.66-0.80), 0.80 (95% CI, 0.70-0.88), and 0.75 (95% CI, 0.67-0.83), respectively. The model showed improved discrimination compared with the National Amyloidosis Centre and Mayo Clinic staging systems, with Harrell concordance indices 2% to 10% higher and 3-year AUC improvements ranging from 3% to 19% across cohorts and scoring systems. Calibration was generally acceptable across cohorts and time points. Model performance remained satisfactory in the subgroup of patients receiving disease-modifying therapy. Explainability analyses identified clinically plausible drivers of risk.

**Conclusions and Relevance:**

An ML-based time-to-event prediction model demonstrated promising predictive performance and showed improved discrimination compared with established staging systems in patients with ATTR-CM, supporting its potential for individualized prognostication.

## Key Points

**Question:**

Can a machine learning–based model improve risk prediction in patients with transthyretin cardiac amyloidosis (ATTR-CM)?

**Findings:**

In this multicenter cohort study including 850 patients with ATTR-CM, a machine learning–based time-to-event model demonstrated better risk discrimination for a composite end point of all-cause mortality and hospitalization for heart failure than the National Amyloidosis Centre and Mayo Clinic staging systems.

**Meaning:**

These findings suggest that machine learning–based risk prediction could potentially improve individualized prognostic assessment in patients with ATTR-CM.

## Teaser

This cohort study develops and validates a machine learning–based prediction model for patients with transthyretin cardiac amyloidosis (ATTR-CM).

## Introduction

Transthyretin cardiac amyloidosis (ATTR-CM) is a progressive infiltrative cardiomyopathy caused by destabilization of transthyretin tetramers that dissociate into monomers, ultimately aggregating into amyloid fibrils.^1^ Myocardial fibril accumulation increases ventricular stiffness and leads to diastolic dysfunction, heart failure (HF), and arrhythmias.^2^ As the disease progresses, patients face a substantial risk of cardiovascular events and death.^1^

In untreated cohorts, median survival ranges from approximately 20 to 66 months,^3^ depending on disease stage. The introduction of disease-modifying therapies has fundamentally altered the natural course of ATTR-CM, offering a demonstrable survival benefit and inaugurating a new therapeutic era.^4^ Nevertheless, clinicians still lack a widely validated and contemporary risk model capable of accurately stratifying prognosis at diagnosis, an essential step in guiding treatment decisions and patient follow-up.

Among the classic prognostic tools, the Mayo Clinic^5^ and National Amyloidosis Centre (NAC)^6^ staging systems remain the most widely adopted. Both rely on cardiac biomarkers and kidney function to stratify patients into prognostic groups, with good performance reported in their cohorts.^5,6^ However, both systems share important limitations: they were developed before the widespread adoption of disease-modifying therapies and may not fully capture risk trajectories in contemporary treated populations.^7^

Given the paradigm shift of disease-modifying therapy, several groups have sought to refine prognostication in ATTR-CM. A retrospective analysis of tafamidis-treated patients showed that traditional NAC and Mayo Clinic staging systems retain only partial discriminative ability in this therapeutic era.^8^ Recognizing that the mortality benefit of tafamidis becomes apparent mainly after 18 months of treatment, researchers proposed a simple clinical score, based on age, cardiac biomarkers, and Eastern Cooperative Oncology Group performance status, to identify patients at risk of early death in wild-type transthyretin amyloidosis (ATTRwt).^9^

Beyond traditional statistics, artificial intelligence (AI) applications have begun to show diagnostic and prognostic potential in amyloidosis.^10^ The A2E score, an AI-enhanced electrocardiogram model estimating the likelihood of cardiac amyloidosis, has been associated with more than a 2-fold increase in the risk of all-cause death.^11^ However, this model was not developed to predict outcomes, nor was it tailored to ATTR-CM, limiting its applicability for comprehensive cardiovascular prognostication in this population.

Despite growing interest, no dedicated AI-based prognostic model in ATTR-CM has yet been developed.^12^ Existing risk scores rely solely on serum biomarkers and conventional clinical parameters, leaving substantial room for improvement.^13^ Developing such a tool represents a major unmet clinical need to guide therapeutic decision-making and improve outcomes in this rapidly evolving field. Therefore, in this study, we sought to develop and validate an AI-based model to predict the composite end point of all-cause mortality and hospitalization for HF in patients with ATTR-CM, leveraging multicenter clinical, biomarker, and imaging data.

## Methods

### Study Design, Population, and Data Collection

eFigure 1 in Supplement 1 presents a summary of the current study. We prospectively included patients from the Swiss Cardiac Amyloidosis Registry (ClinicalTrials.gov identifier: NCT04776824) with a confirmed diagnosis of ATTR-CM. Patients were enrolled across 6 tertiary centers in Switzerland (Inselspital Bern, Luzerner Kantonsspital, Stadtspital Triemli Zürich, Kantonsspital St. Gallen, Centre Hospitalier Universitaire Vaudois Lausanne, and Universitätsspital Basel) between February 2018 and November 2025. Patients from the Cardiac Amyloidosis Registry at the Medical University of Vienna were included based on the same criteria from July 2014 to February 2025. Diagnosis followed standardized criteria: following exclusion of light-chain amyloidosis, ATTR-CM was confirmed by either technetium-labeled bone scintigraphy and/or endomyocardial biopsy. Genetic testing was offered to all patients to differentiate ATTRwt from variant transthyretin amyloidosis. Patients with coexisting light-chain amyloidosis and ATTR amyloidosis were included only if ATTR was deemed the clinically predominant disease and the light-chain amyloidosis component was consistent with localized amyloidosis not requiring systemic therapy. We collected baseline characteristics, including clinical, laboratory, imaging, and genetic testing results. Data analysis was conducted from December 2025 to February 2026.

### Ethics Approval

The Swiss and Vienna registries were approved by the Bern cantonal ethics committee and the Ethics Committee of the Medical University of Vienna, respectively. The study was conducted in compliance with the Declaration of Helsinki. Participants provided written informed consent.

### Outcome Definitions

Systematic follow-up evaluations began 6 months after diagnosis and were conducted annually thereafter. The primary outcome was defined as a composite of all-cause mortality and hospitalization for HF. Each event was prospectively recorded during structured follow-up visits and verified through clinical records, hospitalization reports, and death notifications when available.

### Data Preprocessing

Baseline was defined as the time of diagnosis; when complete data were unavailable at that time point (27 patients; median [IQR] delay, 136 [95-257] days), the first subsequent visit with complete information was used. For each patient, we extracted all available information at baseline, encompassing all features with established or plausible clinical relevance in ATTR-CM as informed by the existing literature. To minimize missing data, any echocardiography or laboratory measurements still unavailable at baseline were replaced by the closest available value within a 6-month window before or after, provided no event had occurred in between. Features with a missing rate of more than 20% were excluded from the analysis.

### Machine Learning Model Development and Evaluation

Time-to-event modeling was performed using a random survival forest (RSF) model, evaluated through an internal-external cross-validation approach in which each center was iteratively held out for validation (untouched test set) while the model was developed on the remaining centers. Three cohorts were defined: Bern, Switzerland (Bern-Swiss cohort), the other Swiss centers combined (Other-Swiss cohort), and Vienna, Austria (Vienna cohort). At each fold, the following steps were repeated on the training data: missing data imputation with the MissForest algorithm, least absolute shrinkage and selection operator (LASSO)–based feature selection, hyperparameter tuning, and RSF training.

Discrimination was evaluated using the Harrell, Uno, and Antolini concordance indices (C indices) and time-dependent area under the curve (AUC) at 1, 2, and 3 years and was compared with the NAC and Mayo Clinic staging systems in the subpopulations with complete data for score computation. Calibration was assessed graphically using smooth calibration curves derived from a flexible adaptive hazard regression model^14^ and quantitatively using the integrated calibration index, mean calibration (ratio of observed to predicted survival), and calibration slope.

Clinical utility was evaluated through decision curve analysis, and model interpretability was assessed using the SurvSHAP algorithm.^15^ Patients were stratified into tertiles of the predicted 3-year risk score, defining low-, medium-, and high-risk groups, and Kaplan-Meier curves were constructed for comparison with the NAC and Mayo Clinic staging groups. For comparison, a Cox proportional hazards model with LASSO regularization was trained using the same pipeline as the RSF. Following cross-validation evaluation, a final model was trained on the entire pooled cohort and deployed as a freely available web application.

Model development and hyperparameter tuning were performed with scikit-learn and scikit-survival packages in Python 3.10 (Python Software Foundation). Calibration curves and statistical tests were performed with R version 4.2 (R Foundation).

### Statistical and Sensitivity Analyses

Baseline characteristics are presented for the entire population and separately by cohort. The subgroups were compared using χ^2^ tests for categorical variables and Mann-Whitney *U* tests for continuous variables. Categorical features are reported as frequencies with percentages, while continuous features are presented as medians with IQRs. Median follow-up was estimated using the inverse Kaplan-Meier method. For all performance metrics, SEs and corresponding 95% CIs were estimated using 1000 bootstrap iterations. The model was further evaluated on the subset of patients receiving amyloid-specific therapy. Additionally, model performance was evaluated using each individual component of the composite end point as the sole outcome.

## Results

### Study Population

A total of 850 patients with ATTR-CM from 6 Swiss hospitals and the Vienna cohort were included (Bern-Swiss cohort, n = 352; Other-Swiss cohort, n = 260; and Vienna cohort, n = 238). The median (IQR) age was 79 (74-83) years, and 750 patients (88.2%) were male. The median (IQR) N-terminal pro–brain natriuretic peptide (NT-proBNP) concentration was 1738 (827-3244) ng/L, and the median (IQR) estimated glomerular filtration rate (eGFR) was 65 (50-79) mL/min/1.73 m^2^. ATTRwt accounted for 627 of 850 cases (73.8%). During a median (IQR) follow-up of 34 (15-59) months, the composite end point occurred in 302 patients (35.5%). In the Bern-Swiss cohort, the median (IQR) follow-up was 38 (20-56) months with a composite end point incidence of 39.8% (140 of 352 patients), compared with 20 (12-38) months and 20.4% (53 of 260 patients) in the Other-Swiss cohort and 53 (21-82) months and 45.8% (109 of 238 patients) in the Vienna cohort. Baseline characteristics are described in Table 1, while event counts and median follow-up time by cohort are reported in eTable 1 in Supplement 1. The estimated event-free survival by center is shown in eFigure 2 in Supplement 1. Missing values per feature are reported in eTable 2 in Supplement 1.

**Table 1.** Baseline Clinical and Demographic Characteristics^a^

Characteristic	Overall (N = 850)	Cohort			*P* value
		Bern-Swiss (n = 352)	Other-Swiss (n = 260)	Vienna (n = 238)	
Amyloidosis subtype, No. (%)					
TTR wild type	627 (73.8)	247 (70.2)	172 (66.2)	208 (87.4)	<.001
TTR unspecified	173 (20.4)	92 (26.1)	81 (31.2)	0	
TTR variant	38 (4.5)	13 (3.7)	5 (1.9)	20 (8.4)	
TTR + AL	12 (1.4)	0	2 (0.8)	10 (4.2)	
Age at baseline, median (IQR)	79 (74-83)	79 (74-83)	78 (74-82)	79 (74-83)	.11
Sex, No. (%)					
Female	100 (11.8)	32 (9.1)	28 (10.8)	40 (16.8)	.03
Male	750 (88.2)	320 (90.9)	232 (89.2)	198 (83.2)	
BMI, median (IQR)	26 (24-28)	26 (24-28)	26 (24-29)	25 (24-28)	.47
NYHA class, No. (%)					
I	143 (17.2)	63 (18.3)	36 (14.2)	44 (19.1)	<.001
II	482 (58.1)	196 (56.8)	176 (69.3)	110 (47.8)	
III	192 (23.2)	77 (22.3)	40 (15.7)	75 (32.6)	
IV	12 (1.4)	9 (2.6)	2 (0.8)	1 (0.4)	
NAC score, No. (%)					
I	488 (65.2)	209 (66.8)	142 (71.7)	137 (57.8)	<.001
II	160 (21.4)	67 (21.4)	36 (18.2)	57 (24.1)	
III	67 (9.0)	22 (7.0)	14 (7.1)	31 (13.1)	
IV	33 (4.4)	15 (4.8)	6 (3.0)	12 (5.1)	
Mayo Clinic score, No. (%)					
I	309 (52.6)	147 (53.1)	66 (60.0)	96 (48.0)	<.001
II	165 (28.1)	75 (27.1)	28 (25.5)	62 (31.0)	
III	113 (19.3)	55 (19.9)	16 (14.5)	42 (21.0)	
Laboratory values, median (IQR)					
NT-proBNP, pg/mL	1738 (827-3244)	1699 (846-3197)	1528 (804-2900)	1929 (839-3785)	.08
Creatinine, mg/dL	1.11 (0.94-1.38)	1.11 (0.96-1.38)	1.11 (0.92-1.27)	1.12 (0.95-1.41)	.40
eGFR, mL/min/1.73 m^2^^b^	65 (50-79)	67 (51-82)	68 (57-81)	61 (45-74)	<.001
Potassium, mEq/L	4 (4-5)	4 (4-4)	4 (4-5)	4 (4-5)	<.001
Sodium, mEq/L	140 (138-141)	140 (138-142)	140 (139-141)	139 (137-141)	<.001
Hemoglobin, g/dL	14.0 (12.7-14.9)	14.0 (12.6-15.0)	14.1 (13.2-14.8)	13.8 (12.4-14.8)	.19
Echocardiography results, median (IQR)					
LVEF, %	55 (46-61)	55 (48-61)	55 (46-61)	52 (45-61)	.11
LVEDD, mm	43 (39-48)	44 (40-49)	44 (40-49)	42 (37-46)	<.001
Maximum wall thickness, mm	17 (15-19)	16 (14-18)	16 (15-19)	19 (17-22)	<.001
Comorbidities, No. (%)					
History of valvular heart disease	138 (16.2)	77 (21.9)	31 (11.9)	30 (12.6)	<.001
Aortic valve disease > grade I	126 (14.8)	86 (24.4)	23 (8.8)	17 (7.1)	<.001
Mitral valve disease > grade I	278 (32.7)	128 (36.4)	37 (14.2)	113 (47.5)	<.001
Tricuspid valve disease > grade I	223 (26.2)	101 (28.7)	26 (10.0)	96 (40.3)	<.001
AF or AFL	465 (54.7)	190 (54.0)	143 (55.0)	132 (55.5)	.93
Pacemaker implanted	79 (9.3)	42 (11.9)	32 (12.3)	5 (2.1)	<.001
Arterial hypertension	502 (59.1)	200 (56.8)	142 (54.6)	160 (67.2)	<.001
Diabetes	165 (19.4)	80 (22.7)	36 (13.8)	49 (20.6)	.01
History of cancer	187 (22.0)	78 (22.2)	45 (17.3)	64 (26.9)	.04
Medical therapy, No. (%)					
Amyloid-specific therapy	539 (63.4)	201 (57.1)	181 (69.6)	157 (66.0)	.004
β-Blocker therapy	409 (48.1)	167 (47.4)	119 (45.8)	123 (51.7)	.40
RAAS inhibitor therapy	498 (58.6)	205 (58.2)	179 (68.8)	114 (47.9)	<.001
MRA therapy	224 (26.4)	85 (24.1)	39 (15.0)	100 (42.0)	<.001
SGLT2 inhibitor therapy	218 (25.6)	82 (23.3)	85 (32.7)	51 (21.4)	.01
Diuretic therapy	496 (58.4)	216 (61.4)	151 (58.1)	129 (54.2)	.22

Abbreviations: AF, atrial fibrillation; AFL, atrial flutter; AL, light-chain amyloidosis; BMI, body mass index (calculated as weight in kilograms divided by height in meters squared); eGFR, estimated glomerular filtration rate; LVEDD, left ventricular end-diastolic diameter; LVEF, left ventricular ejection fraction; MRA, mineralocorticoid receptor antagonist; NAC, National Amyloidosis Centre; NT-proBNP, N-terminal pro–brain natriuretic peptide; NYHA, New York Heart Association; RAAS, renin-angiotensin-aldosterone system; SGLT2, sodium-glucose cotransporter 2; TTR, transthyretin.

SI conversion factors: To convert creatinine to micromoles per liter, multiply by 88.4; potassium and sodium to millimoles per liter, multiply by 1; and hemoglobin to grams per liter, multiply by 10.

^a^ Descriptive statistics of the population are shown overall and stratified by cohort. *P* values for comparisons between the 3 cohorts are reported.

^b^ Calculated using the Chronic Kidney Disease Epidemiology Collaboration equation.

### Model Discrimination

The discrimination metrics of the RSF model are reported in Table 2. The RSF model achieved Harrell C indices of 0.74 (95% CI, 0.69-0.78), 0.77 (95% CI, 0.69-0.83), and 0.72 (95% CI, 0.67-0.77) in the Bern-Swiss, Other-Swiss, and Vienna cohorts, respectively. When compared with established staging systems in the subpopulations with available scores (details in eTable 3 in Supplement 1), the RSF model demonstrated higher discrimination compared with the NAC and Mayo Clinic staging systems (Table 3). Harrell C indices for the RSF model ranged from 0.72 to 0.78 across cohorts, compared with 0.65 to 0.74 for the NAC staging system and 0.66 to 0.70 for the Mayo Clinic staging system, with improvements ranging from 2% to 10% across cohorts. This pattern was consistent when discrimination was assessed by 3-year AUC, with the RSF model showing better performance than both staging systems across all cohorts. The 3-year AUC for the RSF model ranged from 0.74 (95% CI, 0.66-0.81) to 0.86 (95% CI, 0.72-0.97) across cohorts, compared with 0.62 (95% CI, 0.55-0.69) to 0.70 (95% CI, 0.58-0.81) for the NAC staging system and 0.65 (95% CI, 0.57-0.73) to 0.72 (95% CI, 0.64-0.80) for the Mayo Clinic staging system, with improvements ranging from 3% to 19% compared with the Mayo Clinic staging system and from 5% to 12% compared with the NAC staging system.

**Table 2.** Discrimination and Calibration Performance of the Random Survival Forest Model, Evaluated by Internal-External Cross-Validation

Measure	Cohort		
	Bern-Swiss	Other-Swiss	Vienna
Discrimination			
Concordance index (95% CI)			
Harrell	0.74 (0.69-0.78)	0.77 (0.69-0.83)	0.72 (0.67-0.77)
Uno	0.71 (0.66-0.76)	0.75 (0.68-0.82)	0.71 (0.65-0.77)
Antolini	0.74 (0.70-0.78)	0.76 (0.68-0.83)	0.73 (0.68-0.78)
AUC (95% CI)			
1 y	0.81 (0.74-0.87)	0.80 (0.70-0.89)	0.78 (0.69-0.85)
2 y	0.75 (0.68-0.81)	0.74 (0.65-0.83)	0.73 (0.65-0.80)
3 y	0.73 (0.66-0.80)	0.80 (0.70-0.88)	0.75 (0.67-0.83)
Calibration			
Mean calibration (95% CI)			
1 y	0.88 (0.64-1.13)	1.26 (0.85-1.63)	0.95 (0.68-1.25)
2 y	1.09 (0.88-1.28)	1.02 (0.76-1.30)	0.91 (0.71-1.10)
3 y	1.14 (0.98-1.32)	0.78 (0.58-0.99)	0.98 (0.83-1.14)
Slope (95% CI)			
1 y	0.94 (0.73-1.18)	0.96 (0.64-1.37)	0.78 (0.56-1.08)
2 y	1.04 (0.79-1.30)	1.14 (0.75-1.62)	0.86 (0.61-1.17)
3 y	1.04 (0.79-1.32)	1.43 (1.00-1.99)	0.98 (0.69-1.33)
ICI (95% CI)			
1 y	0.03 (0.01-0.05)	0.018 (0.004-0.047)	0.02 (0.01-0.06)
2 y	0.05 (0.01-0.08)	0.01 (0.01-0.06)	0.02 (0.01-0.07)
3 y	0.04 (0.01-0.09)	0.05 (0.02-0.11)	0.02 (0.01-0.08)

Abbreviations: AUC, area under the curve; ICI, integrated calibration index.

**Table 3.** Discrimination Performance of the RSF Model Compared With the Mayo Clinic and NAC Staging Scores^a^

Model	Concordance index (95% CI)			AUC (95% CI)		
	Harrell	Uno	Antolini	1 y	2 y	3 y
**Mayo Clinic staging system**						
Bern-Swiss cohort						
Score	0.66 (0.60-0.72)	0.64 (0.58-0.70)	0.66 (0.60-0.72)	0.71 (0.60-0.80)	0.67 (0.59-0.75)	0.65 (0.57-0.73)
RSF	0.76 (0.71-0.80)	0.73 (0.68-0.78)	0.76 (0.71-0.80)	0.81 (0.73-0.88)	0.76 (0.69-0.83)	0.75 (0.68-0.82)
Other-Swiss cohort						
Score	0.70 (0.58-0.83)	0.73 (0.60-0.85)	0.70 (0.58-0.83)	0.68 (0.53-0.83)	0.66 (0.50-0.82)	0.67 (0.48-0.84)
RSF	0.78 (0.65-0.89)	0.76 (0.64-0.87)	0.79 (0.68-0.89)	0.82 (0.68-0.94)	0.71 (0.55-0.87)	0.86 (0.72-0.97)
Vienna cohort						
Score	0.70 (0.64-0.75)	0.69 (0.62-0.74)	0.70 (0.64-0.75)	0.73 (0.62-0.81)	0.69 (0.60-0.78)	0.72 (0.64-0.80)
RSF	0.72 (0.64-0.79)	0.70 (0.62-0.78)	0.72 (0.65-0.80)	0.75 (0.63-0.86)	0.75 (0.64-0.84)	0.75 (0.64-0.85)
**NAC staging system**						
Bern-Swiss cohort						
Score	0.65 (0.59-0.70)	0.63 (0.57-0.68)	0.65 (0.59-0.70)	0.70 (0.60-0.79)	0.66 (0.58-0.73)	0.62 (0.55-0.69)
RSF	0.75 (0.71-0.79)	0.72 (0.67-0.77)	0.75 (0.71-0.79)	0.81 (0.74-0.88)	0.75 (0.67-0.82)	0.74 (0.66-0.81)
Other-Swiss						
Score	0.74 (0.64-0.83)	0.69 (0.59-0.79)	0.74 (0.64-0.83)	0.76 (0.64-0.88)	0.70 (0.59-0.81)	0.70 (0.58-0.81)
RSF	0.78 (0.70-0.86)	0.76 (0.67-0.84)	0.78 (0.70-0.86)	0.83 (0.72-0.93)	0.74 (0.63-0.85)	0.80 (0.69-0.90)
Vienna						
Score	0.67 (0.62-0.71)	0.67 (0.61-0.72)	0.67 (0.62-0.72)	0.72 (0.63-0.81)	0.68 (0.60-0.75)	0.70 (0.63-0.78)
RSF	0.72 (0.64-0.79)	0.71 (0.63-0.79)	0.73 (0.66-0.80)	0.74 (0.61-0.86)	0.75 (0.64-0.84)	0.75 (0.64-0.85)

Abbreviations: AUC, area under the curve; NAC, National Amyloidosis Centre; RSF, random survival forest.

^a^ As staging scores were not available for all patients, metrics for each score are reported on the corresponding subcohort of patients with available data.

### Model Calibration

**Figure 1.**
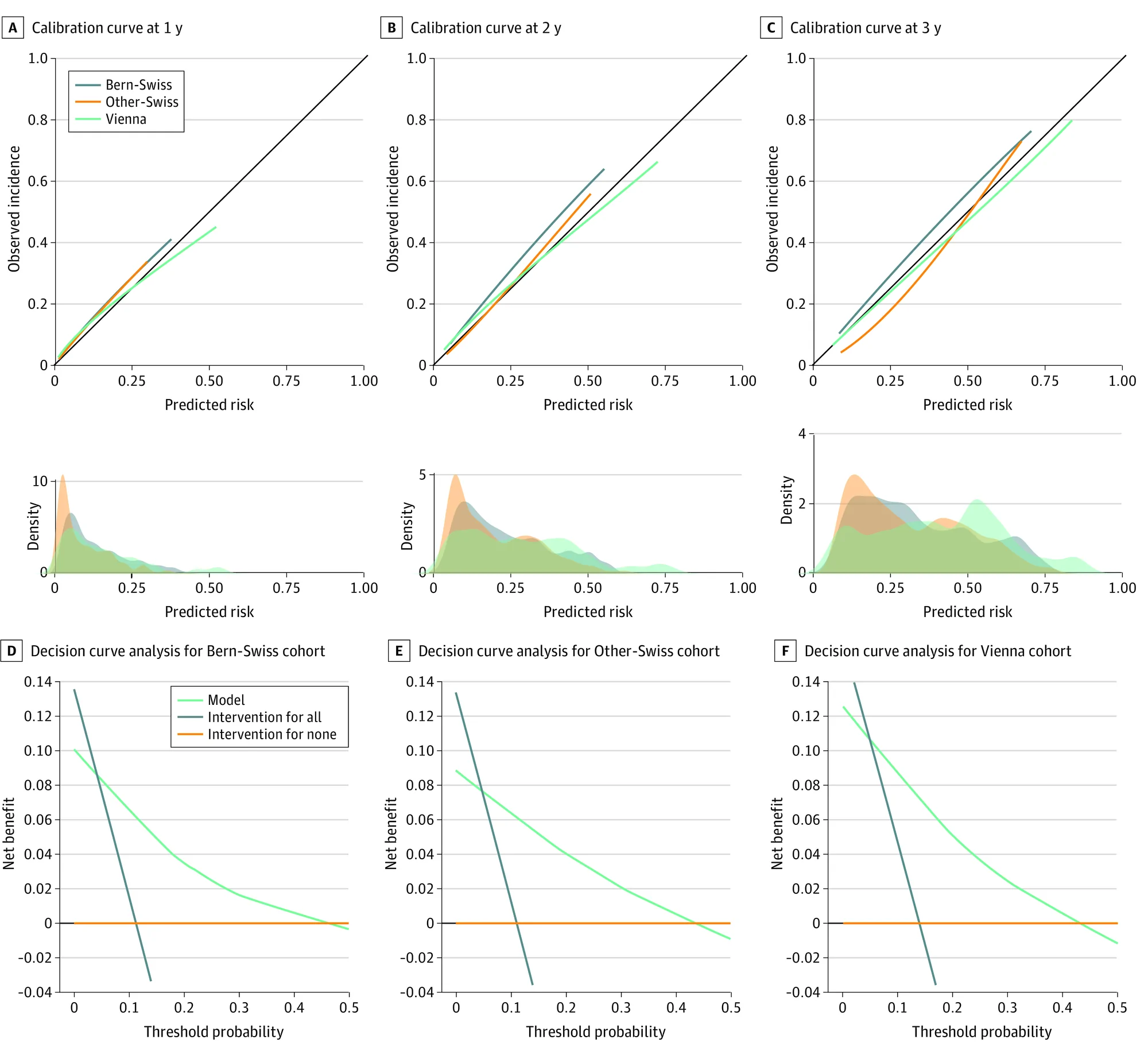
Calibration Curves and Decision Curve Analysis of the Clinical Utility of the Random Survival Forest (RSF) Model Across Datasets Calibration curves are shown for RSF predicted risk at 1 year (A), 2 years (B), and 3 years (C) across datasets, with the lower panel of each showing the distribution of predicted values. Decision curve analysis is shown across datasets for the Bern-Swiss (D), Other-Swiss (E), and Vienna (F) cohorts. The net benefit of the RSF model at 1 year is compared with the strategies of intervening in all patients or in none.

Calibration metrics for the RSF model are reported in Table 2, and calibration curves are shown in Figure 1A-C. Overall, calibration was acceptable across all cohorts, with mean calibration and calibration slope values close to 1 and an integrated calibration index ranging from 0.01 to 0.05 across all 3 time points, indicating good agreement between predicted and observed risk. In the Other-Swiss cohort, calibration was satisfactory at 1 and 2 years but deteriorated at 3 years, as reflected by a mean calibration of 0.78 (95% CI, 0.58-0.99) and a calibration slope of 1.43 (95% CI, 1.00-1.99), suggesting a tendency to overestimate risk at longer time horizons. This is partly expected given the shorter median follow-up in this cohort, which limits the reliability of long-term calibration estimates. These findings are further supported by the calibration curves, which demonstrate good overall agreement between predicted and observed risk.

### Decision Curve Analysis

The decision curve analysis of the RSF model’s 1-year net benefit across different thresholds is shown in Figure 1D-F. The model is compared with the naive strategies of intervening for all patients and for none. The RSF model achieved a higher net benefit than the other 2 strategies across a wide range of clinically relevant thresholds. For example, at a risk threshold of 15% (which approximately corresponds to the cutoff for the high-risk class), the net benefit in the Bern-Swiss, Other-Swiss, and Vienna cohorts was 0.052, 0.052, and 0.059, respectively, corresponding to approximately 5, 5, and 6 additional true events identified per 100 patients compared with a strategy of intervening in none. Similar results were observed at the 2- and 3-year time points (eFigure 3 in Supplement 1).

### Explainability

**Figure 2.**
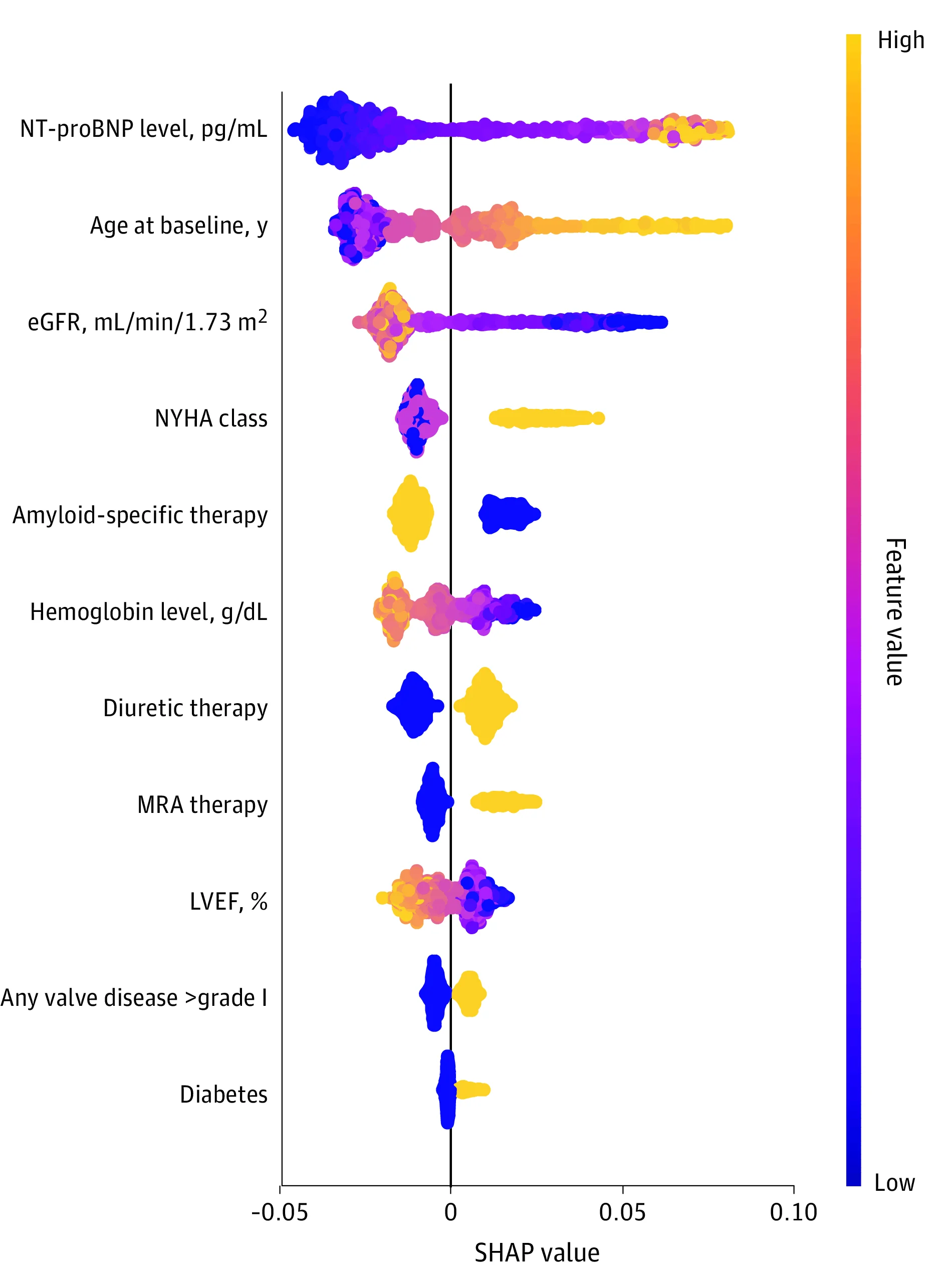
SurvSHAP Summary Plot of the Global Explainability of the Random Survival Forest A SurvSHAP summary plot shows the global explainability of the random survival forest model trained on the combined dataset from all 3 cohorts. SurvSHAP values are computed on the entire pooled cohort. Each dot represents an individual patient, with color indicating the corresponding feature value. The x-axis shows the SurvSHAP value, reflecting the contribution of each feature to the model’s prediction; positive values indicate increased risk, while negative values indicate reduced risk. NT-proBNP indicates N-terminal pro–brain natriuretic peptide; eGFR, estimated glomerular filtration rate; NYHA, New York Heart Association; MRA, mineralocorticoid receptor antagonist; LVEF, left ventricular ejection fraction; SHAP, Shapley additive explanations.

Figure 2 shows the SurvSHAP summary plot for the final RSF model trained on the entire pooled cohort. The most relevant features were NT-proBNP level, age, eGFR, New York Heart Association (NYHA) class, and amyloid-specific therapy. The direction of feature contributions aligns with clinical intuition: higher values of NT-proBNP, NYHA class, and age were associated with increased risk, while higher values of eGFR were associated with decreased risk. Similar explainability plots for the models trained at each fold of the internal-external cross-validation are shown in eFigure 4 in Supplement 1. Local explanations were also analyzed to inspect individual predictions. Two representative cases correctly classified by the machine learning (ML) model but misclassified by the NAC and Mayo Clinic staging systems are presented in eFigure 5 in Supplement 1.

### Secondary Analysis

Secondary analyses, including model evaluation in the treated subgroup, on individual end point components, and comparison with the Cox-LASSO model, are reported in eTables 4 through 6 and the eAppendix in Supplement 1. Briefly, discrimination was consistent across cohorts in the treated subgroup, with a modest decrease in the Bern-Swiss cohort. Performance on individual components was acceptable, with Harrell C indices of 0.69 (95% CI, 0.63-0.76) to 0.74 (95% CI, 0.66-0.82) for hospitalization for HF and 0.76 (95% CI, 0.71-0.81) to 0.79 (95% CI, 0.67-0.89) for all-cause mortality. Compared with the Cox-LASSO model, the RSF model showed marginally better discrimination, with differences of 1% to 2% in Harrell C index and 2% to 5% in 1-year AUC, more pronounced in the treated subgroup (up to 6% and 17%, respectively).

### Risk Stratification

eFigures 6 and 7 in Supplement 1 show Kaplan-Meier event-free survival curves stratified by ML-derived risk categories and NAC and Mayo Clinic staging groups. Both the NAC and Mayo Clinic staging systems tend to classify the majority of patients into the lowest risk category (stage I), limiting their ability to discriminate across risk groups. In contrast, the ML model demonstrated stronger risk stratification, with 3-year event-free survival in the low-risk group ranging from 85% to 91% across cohorts, compared with 68% to 90% for the lowest-risk category of the NAC and Mayo Clinic systems.

## Discussion

In this multicenter cohort study, we developed and validated an ML-based prediction model for a composite event of all-cause mortality and hospitalization for HF in patients with ATTR-CM by integrating clinical assessments, medication information, laboratory measurements, and echocardiography results.

An RSF model was developed and evaluated using an internal-external cross-validation approach across 3 cohorts of patients with ATTR-CM, with a final model trained on the entire pooled cohort for deployment. This approach allows the generalizability and heterogeneity of model performance to be examined across the different cohorts. The model demonstrated good discrimination, as assessed by the Harrell C index and time-dependent AUC over the 3-year evaluation period. Compared with the NAC and Mayo Clinic staging systems,^5,6^ the RSF model showed better discrimination, as indicated by a higher C index and time-dependent AUC.

Overall, the model showed acceptable calibration across cohorts, with good agreement between predicted and observed risk at 1 and 2 years. A reduction in calibration performance was observed at 3 years in the Other-Swiss cohort, likely reflecting the shorter median follow-up and differences in observed long-term survival in this population.

In terms of clinical utility, decision curve analysis demonstrated a higher net benefit across a wide range of decision thresholds compared with the reference strategies of treating all patients or none. The model’s predictions could therefore guide the identification of patients requiring closer follow-up, including more frequent clinical assessments, home monitoring of body weight, and timely adjustment of diuretic therapy.

The contribution of features to the model’s predictions was explored using SurvSHAP analysis. Variables with the highest prognostic relevance reflected disease burden (NT-proBNP level and NYHA functional class), use of amyloid-specific therapy, and comorbidity burden (age, eGFR, and hemoglobin level). NT-proBNP is a well-established marker of disease progression and severity in ATTR-CM and is already incorporated in the NAC and Mayo Clinic staging systems, reflecting increased myocardial wall stress secondary to elevated filling pressures. Kidney dysfunction is also a well-recognized risk factor in cardiac amyloidosis^6^ and has been studied in detail.^16,17^

Recent efforts have focused on developing AI-based models for diagnosing cardiac amyloidosis, aiming to improve disease recognition and reduce diagnostic delays by integrating clinical, electrocardiographic, and echocardiographic data.^10^ To date, however, no AI-based model, to our knowledge, has been specifically proposed to predict clinical outcomes in ATTR-CM.^18,19^ When compared with established prognostic tools, our ML model showed improved discrimination across cohorts. The disease course of patients with ATTR-CM has changed substantially in recent years, mainly due to the introduction of disease-modifying therapies, increased disease awareness, and earlier diagnosis, and traditional risk stratification systems may only partially retain their discriminative ability in contemporary cohorts. Müller et al^8^ demonstrated that the NAC system shows reduced risk discrimination in the modern therapeutic era, particularly among low- and intermediate-risk patients, a finding consistent with our results.

The clinical relevance of an updated risk stratification model lies not only in improved predictive performance but also in the ability to account for disease-modifying therapies. The prognostic benefit of amyloid-specific therapies has been shown to emerge over time, becoming apparent after approximately 18 months for tafamidis,^4^ whereas earlier benefits have been reported for acoramidis.^20^ ML methods may be particularly suited to capture such time-dependent effects, potentially enabling more individualized risk estimation in populations where treatment benefits evolve over time. The model presented in this study showed satisfactory performance in the treated subgroup, supporting its applicability in real-world clinical practice.

All code and trained models have been made publicly available to ensure reproducibility and support further research. In addition, a freely accessible web application was developed to facilitate clinical use.

### Limitations

This study has some limitations. First, the retrospective design introduces inherent limitations, including missing data, population biases, and loss to follow-up.^21^ Consequently, a prospective evaluation will be necessary to fully assess model robustness and clinical applicability. Second, all cohorts originated from Central European countries, potentially limiting generalizability. External validation in geographically and demographically distinct cohorts will be needed to assess transportability. Third, while the cohort size is large given the rarity of the disease, a larger dataset would likely further improve model performance and stability. Fourth, although model interpretability was explored using the SurvSHAP framework, feature contributions should not be interpreted as causal effects.^22^ The model captures statistical associations present in the data rather than underlying causal mechanisms and should not be used to draw causal inferences. Fifth, although the model appeared to maintain stable performance in the subgroup of patients receiving disease-modifying therapy, the relatively short observation time of the cohort remains an important limitation that should be taken into consideration. Finally, analyses were restricted to first events, as data on recurrent HF hospitalizations were not available for all cohorts. Recurrent-event methods should be considered in future studies to fully capture the burden of hospitalization for HF.

## Conclusions

In this multicenter cohort study, we developed and validated an open-source time-to-event ML model for predicting the composite end point of all-cause mortality and hospitalization for HF among patients with ATTR-CM, using multimodal data. The model demonstrated good and stable discriminative performance and provided incremental prognostic value beyond the NAC and Mayo Clinic staging systems. These findings highlight the potential of ML-based risk prediction to improve individualized prognostication in ATTR-CM.

## Notes

Supplement 1. **eAppendix.** Secondary Analyses: Treated Subgroup, Individual Endpoints, and Cox-LASSO Comparison **eFigure 1.** Flowchart of the Study **eFigure 2.** Kaplan-Meier Event-Free Survival Curves Stratified by Center **eFigure 3.** Decision Curve Analysis Across Centers at 2 and 3 Years **eFigure 4.** Global Explainability of the RSF Models for Each Fold **eFigure 5.** Local Interpretability Plot for Selected Cases **eFigure 6.** Kaplan-Meier Event-Free Survival Curves Stratified by RSF-Derived Risk Categories and NAC Staging System **eFigure 7.** Kaplan-Meier Event-Free Survival Curves Stratified by RSF-Derived Risk Categories and Mayo Staging System **eTable 1.** Events Count and Follow-Up Duration **eTable 2.** Missing Values Count per Feature and Cohort **eTable 3.** Baseline Characteristics by Cohort for Patients With Available Staging Scores **eTable 4.** Model Performance in the Treated Subgroup **eTable 5.** Discrimination Performance on Individual Endpoint Components by Cohort **eTable 6.** Cox Model Discrimination and Calibration by Cohort

Supplement 2. Data Sharing Statement
