# Supplementary material for: Machine Learning–Driven Risk Prediction Model in Transthyretin Amyloid Cardiomyopathy
Source: JAMA Cardiol. 2026 Aug 28;11(10):932–41. doi: 10.1001/jamacardio.2026.3496 (PMC13525483; doi:10.1001/jamacardio.2026.3496)
Supplement: Supplementary file 1 [file jamacardiol-e263496-s001.pdf]

Supplemental Online Content

Baj G, Ciocca N, Mohammadi Kazaj P, et al. Machine learning–driven risk prediction model in transthyretin amyloid cardiomyopathy. *JAMA Cardiol*. Published online August 28, 2026. doi:10.1001/jamacardio.2026.3496

eAppendix. Secondary Analyses: Treated Subgroup, Individual Endpoints, and Cox-LASSO Comparison
eFigure 1. Flowchart of the Study
eFigure 2. Kaplan-Meier Event-Free Survival Curves Stratified by Center
eFigure 3. Decision Curve Analysis Across Centers at 2 and 3 Years
eFigure 4. Global Explainability of the RSF Models for Each Fold
eFigure 5. Local Interpretability Plot for Selected Cases
eFigure 6. Kaplan-Meier Event-Free Survival Curves Stratified by RSF-Derived Risk Categories and NAC Staging System
eFigure 7. Kaplan-Meier Event-Free Survival Curves Stratified by RSF-Derived Risk Categories and Mayo Staging System
eTable 1. Events Count and Follow-Up Duration
eTable 2. Missing Values Count per Feature and Cohort
eTable 3. Baseline Characteristics by Cohort for Patients With Available Staging Scores
eTable 4. Model Performance in the Treated Subgroup
eTable 5. Discrimination Performance on Individual Endpoint Components by Cohort
eTable 6. Cox Model Discrimination and Calibration by Cohort

This supplemental material has been provided by the authors to give readers additional information about their work.

© 2026 Baj G et al. *JAMA Cardiology*.

eAppendix. Secondary Analyses: Treated Subgroup, Individual Endpoints, and Cox-LASSO Comparison

When evaluated on the subpopulation of patients receiving amyloid-specific therapy, discrimination performance was consistent with that observed in the whole cohort for the Other-Swiss and Vienna centers, with Harrell's C-index of 0.74 and 0.72 and 3-year AUC of 0.82 and 0.75, respectively (eTable 4 in the Supplement). A modest performance decrease was observed in the Bern-Swiss cohort, where Harrell's C-index dropped from 0.74 to 0.67 and 3-year AUC from 0.73 to 0.67. Calibration in the treated subgroup was generally acceptable in the Other-Swiss and Vienna cohorts. In the Bern-Swiss cohort, calibration was poorer, particularly at 1 year, with a mean calibration of 0.50 (95% CI: 0.22–0.83) and a calibration slope of 0.61 (95% CI: 0.29–1.00), suggesting underestimation of risk in this subgroup.

The model was additionally evaluated using each individual component of the composite endpoint as the sole outcome. Results are reported in eTable 5 in the Supplement. Discrimination was acceptable across both endpoints and cohorts, with Harrell's C-index ranging from 0.69 to 0.74 for HF hospitalization and from 0.76 to 0.79 for all-cause mortality. Confidence intervals were wider, particularly in the Other-Swiss cohort, reflecting the lower number of events when considering individual components.

When compared to the Cox-LASSO model, the RSF model showed marginally better discrimination across all cohorts, with improvements in Harrell's C-index of 1–2% and in 1-year AUC of 2–5% (eTable 6 in the Supplement). These differences were more pronounced in the subgroup of patients receiving ATTR disease-modifying therapy, with improvements of up to 6% in Harrell's C-index and 17% in 1-year AUC. Calibration was comparable between the two models, except in the Swiss cohort where RSF performed better.

© 2026 Baj G et al. *JAMA Cardiology*.

eFigure 1. Flowchart of the Study.

ATTR-CM: Transthyretin Cardiac Amyloidosis Cardiomyopathy, Echo: Echocardiography, SHAP: SHapley Additive exPlanations, AUC: time-dependent area under the curve, NAC: National Amyloidosis Centre.

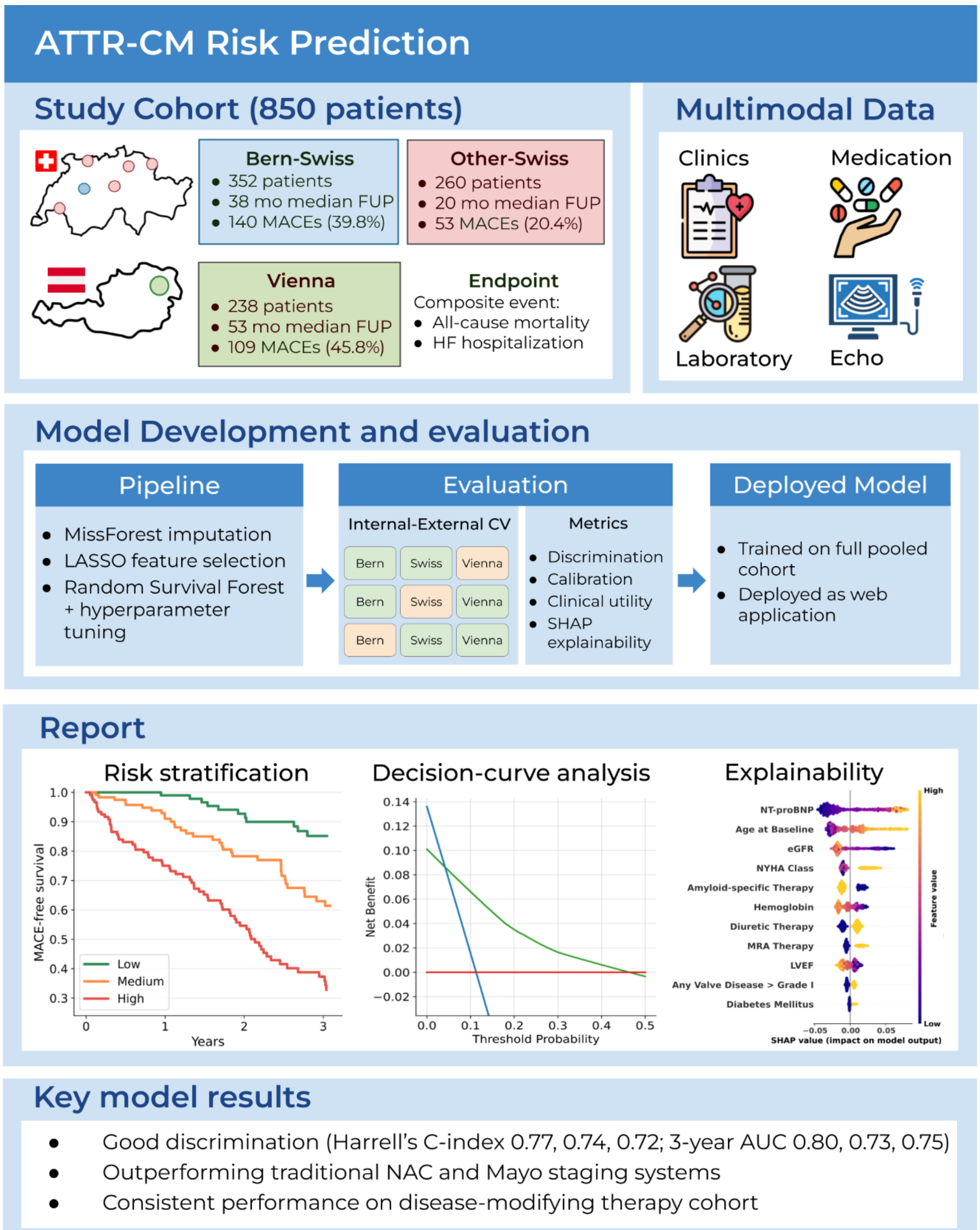

© 2026 Baj G et al. *JAMA Cardiology*.

eFigure 2. Kaplan-Meier Event-Free Survival Curves Stratified by Center

The endpoint is a composite of all-cause mortality and heart-failure hospitalization.

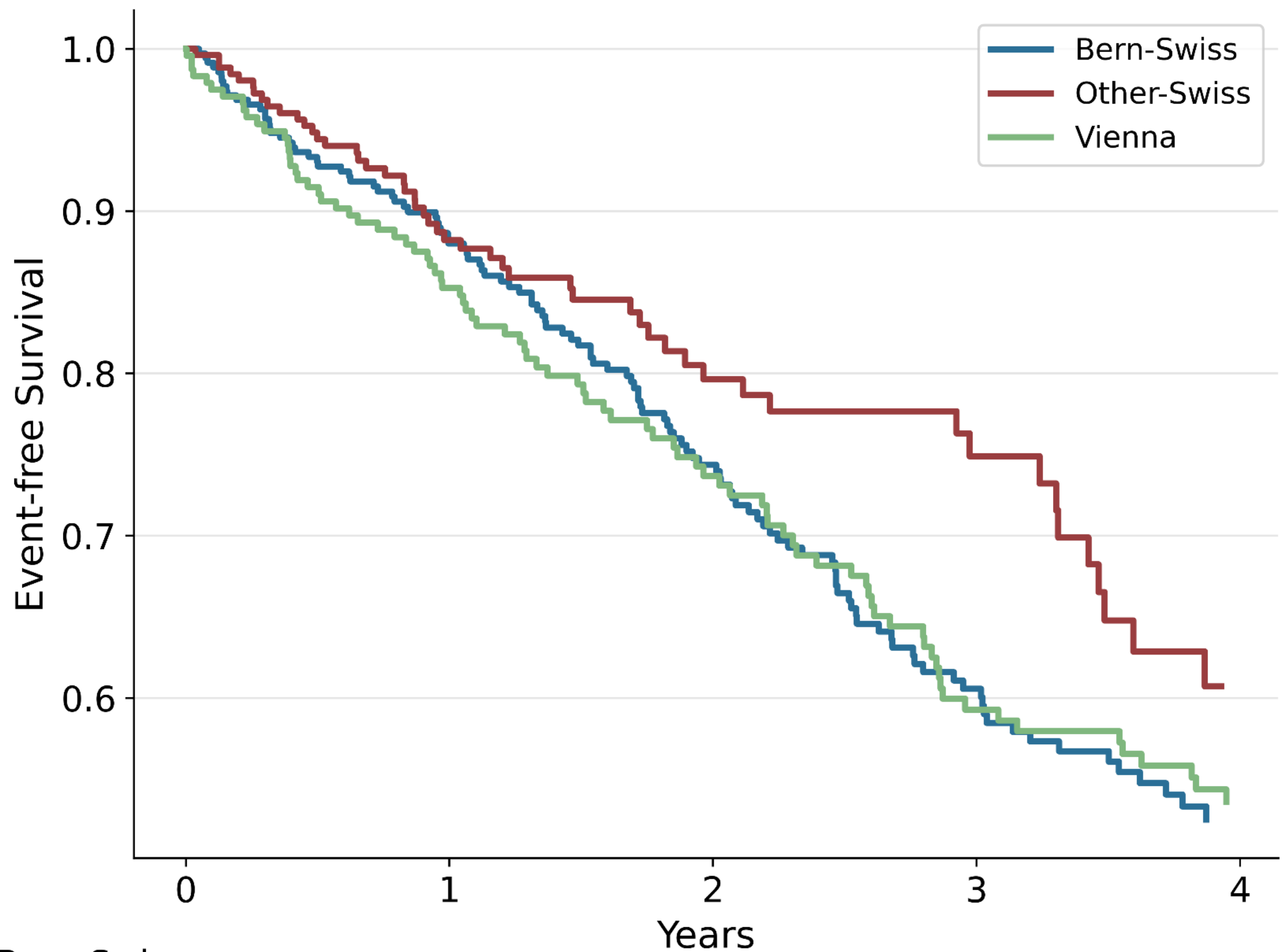

**Bern-Swiss**									
At risk	352	314	272	220	181	142	115	88	66
Censored	0	14	40	74	94	115	130	150	166
Events	0	24	40	58	77	95	107	114	120
**Other-Swiss**									
At risk	260	231	170	125	89	66	52	37	27
Censored	0	15	63	102	132	153	165	174	182
Events	0	14	27	33	39	41	43	49	51
**Vienna**									
At risk	238	210	186	147	124	109	89	83	70
Censored	0	8	18	45	58	64	70	74	81
Events	0	20	34	46	56	65	79	81	87

© 2026 Baj G et al. *JAMA Cardiology*.

eFigure 3. Decision Curve Analysis Across Centers at 2 and 3 Years

The net benefit of the RSF models at 2 and 3 years is compared with the basic strategies of intervening in all patients or in none.

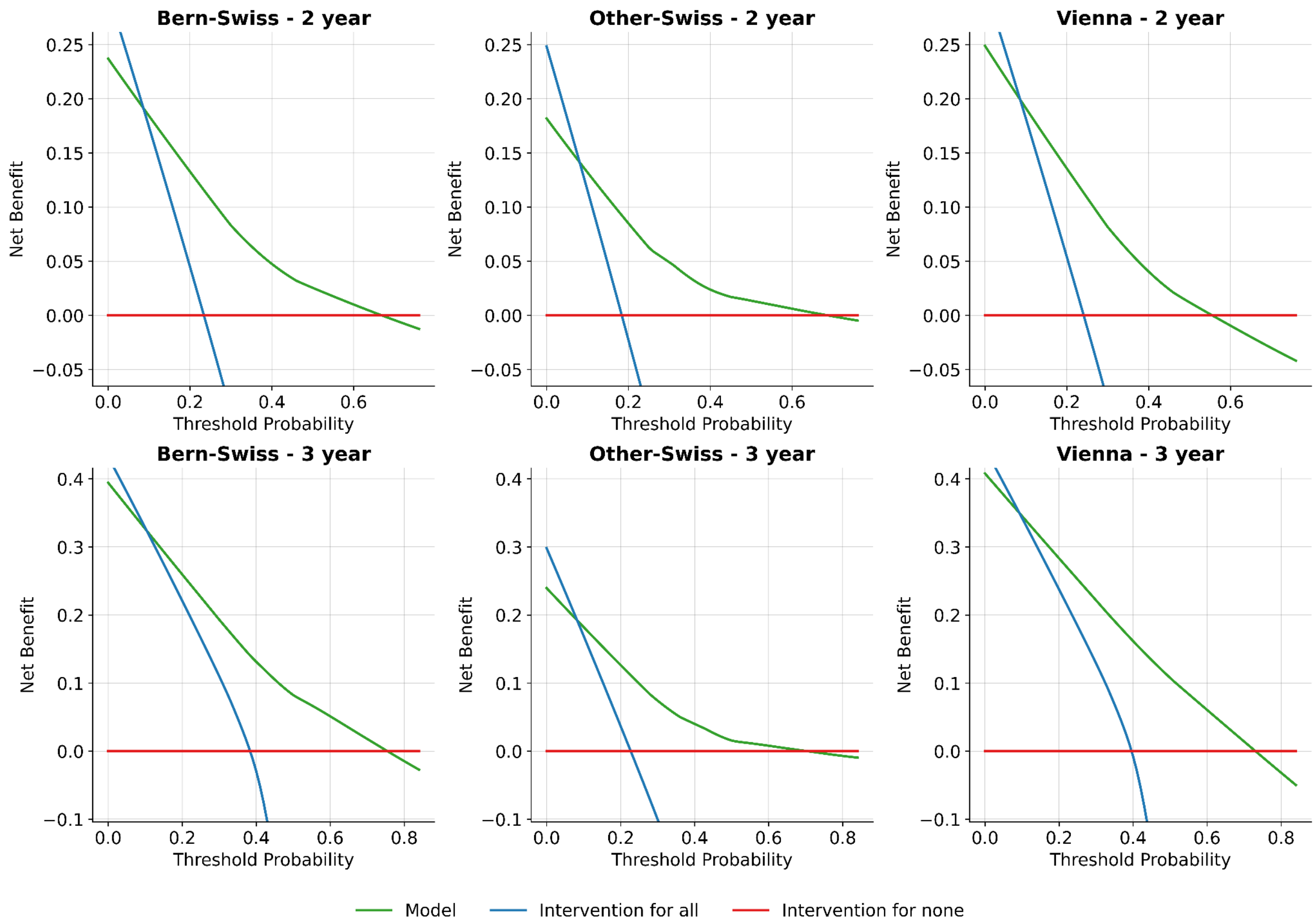

© 2026 Baj G et al. *JAMA Cardiology*.

eFigure 4. Global Explainability of the RSF Models for Each Fold

SurvSHAP summary plots illustrating global explainability of the RSF models trained at each fold of the internal-external cross-validation, shown separately by held-out center (Bern, Swiss combined, and Vienna). As LASSO-based feature selection was repeated independently at each fold, the set of selected features may differ across centers. Each dot represents an individual patient, with color indicating the corresponding feature value. The x-axis shows the SurvSHAP value, reflecting the contribution of each feature to the model's prediction: positive values indicate increased risk, while negative values indicate reduced risk.

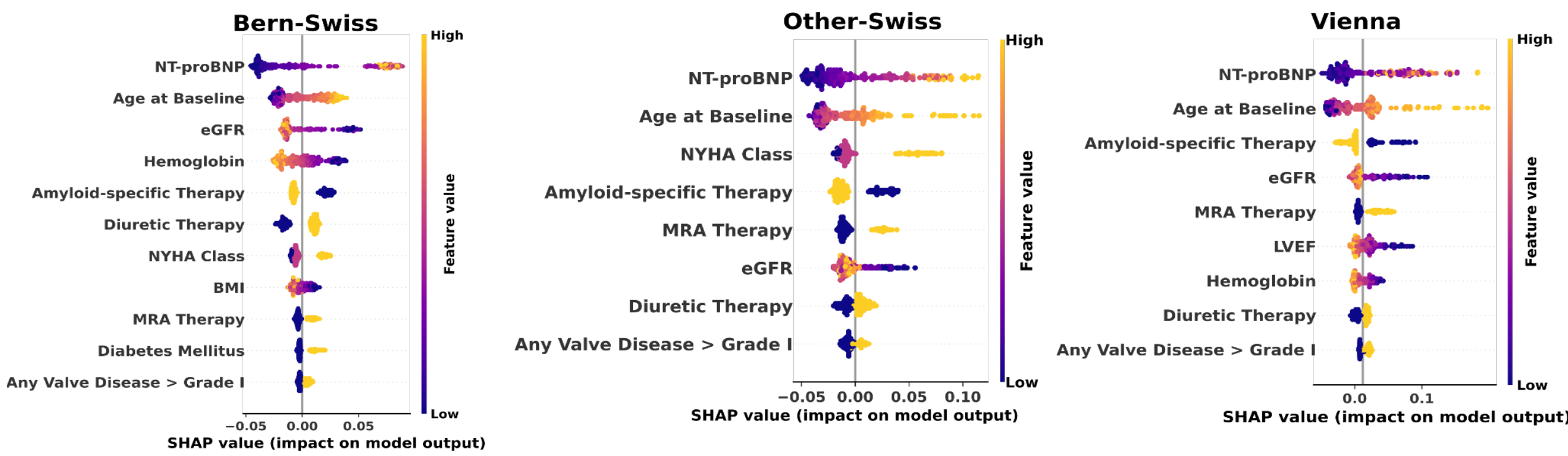

© 2026 Baj G et al. *JAMA Cardiology*.

eFigure 5. Local Interpretability Plot for Selected Cases

Local interpretability plot for selected cases, showing the contribution of individual features to the predicted risk score. Red bars indicate features that increase the predicted risk, while green bars represent features that decrease it. The length of each bar reflects the magnitude of influence. **a)** A 73-year-old patient was identified as low risk by the ML model, but classified in Stage II by the NAC and Mayo staging systems. The patient did not experience any event up to 1332 days after baseline. The features contributing the most to a low risk are NT-proBNP, age, and amyloid-specific therapy. **b)** An 84-year-old patient was classified as high risk by the ML model, but identified as Stage I in both NAC and Mayo systems. The patient died 226 days after baseline. Lower hemoglobin, higher age, amyloid-specific therapy, and diuretic therapy are contributing the most to increasing the risk.

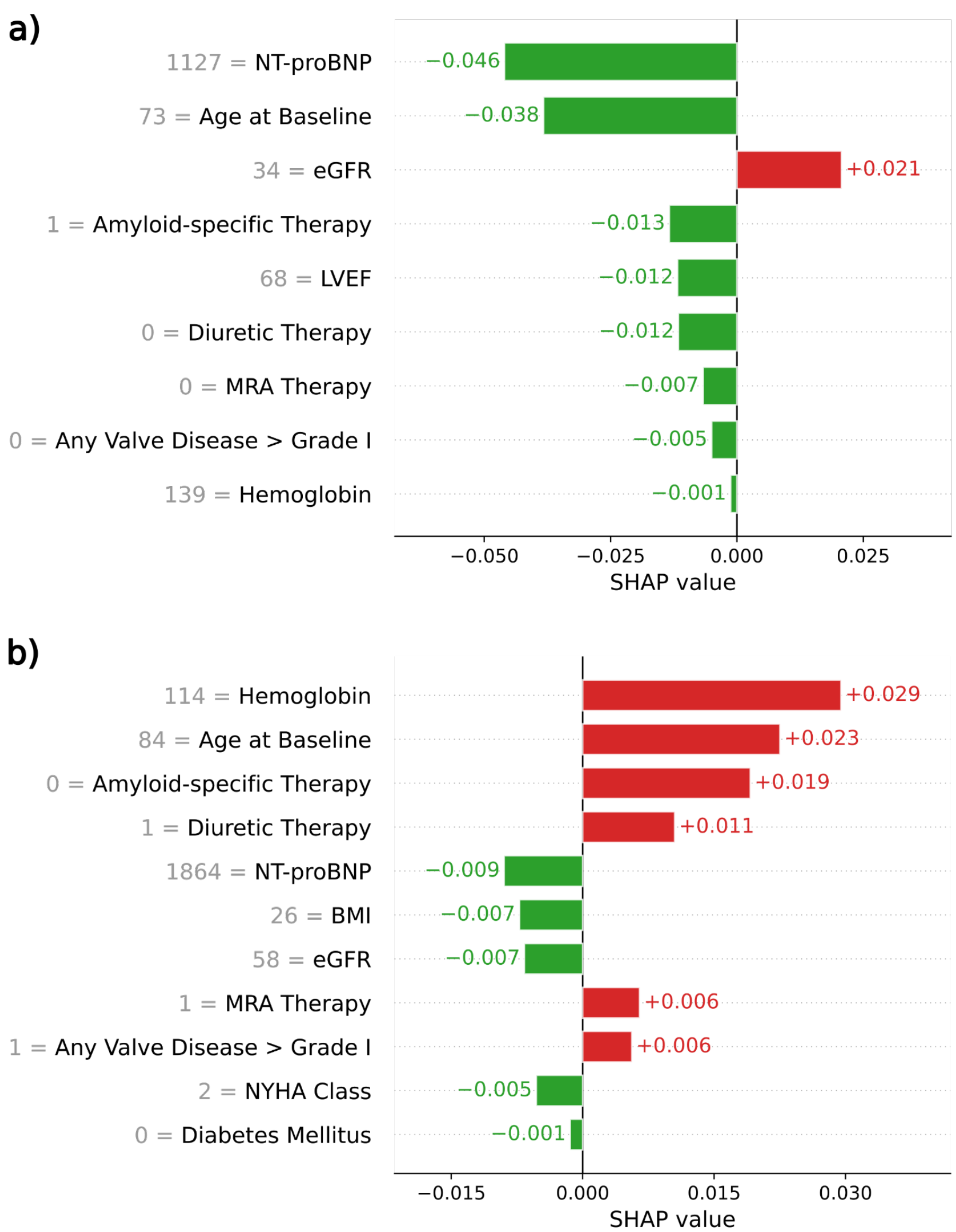

© 2026 Baj G et al. *JAMA Cardiology*.

eFigure 6. Kaplan-Meier Event-Free Survival Curves Stratified by RSF-Derived Risk Categories and NAC Staging System

Kaplan-Meier event-free survival curves for the Bern-Swiss, Other-Swiss, and Vienna cohorts (upper, middle, and lower panels, respectively), stratified by RSF model-derived risk category (left panels) and NAC staging system (right panels).

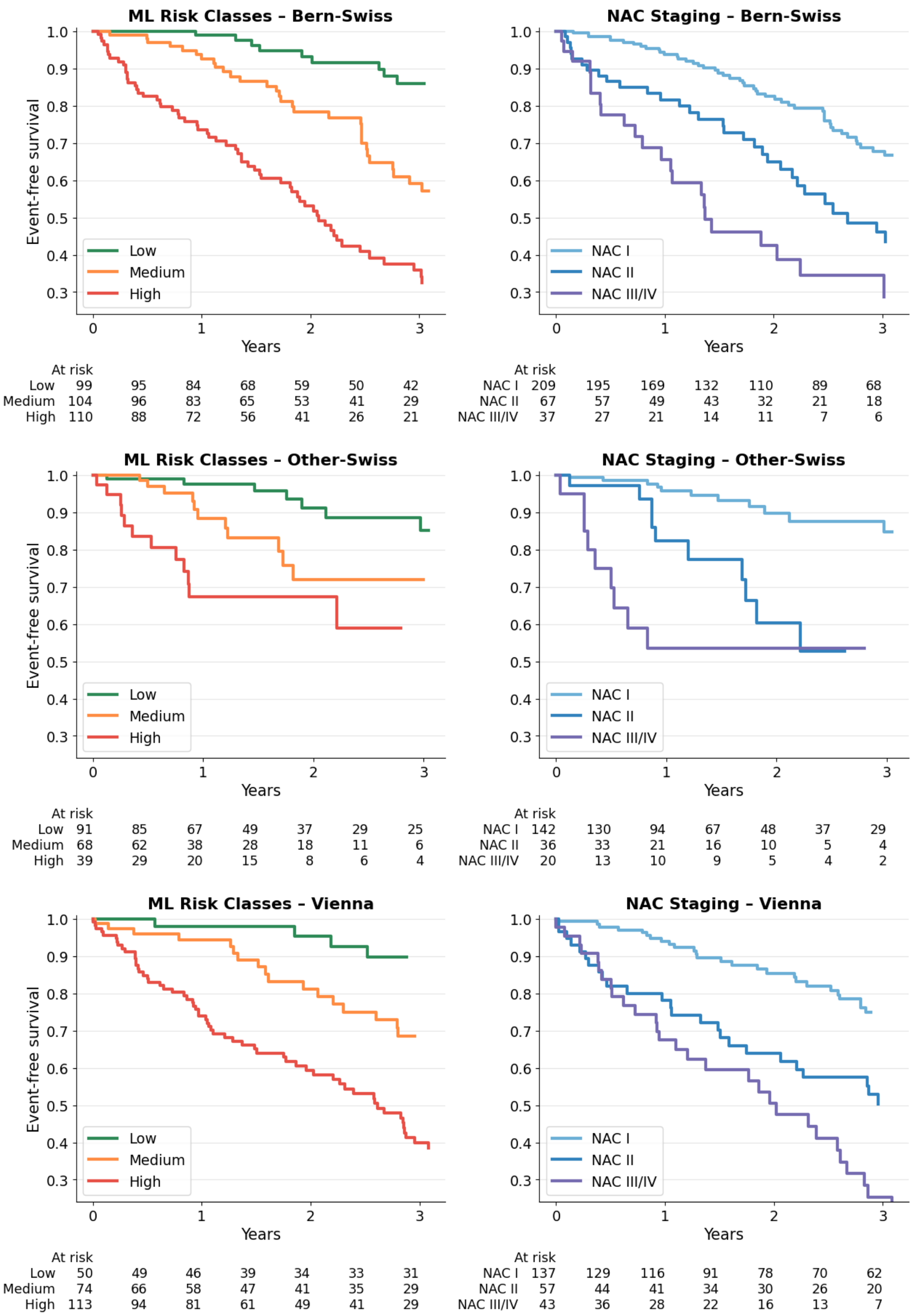

© 2026 Baj G et al. *JAMA Cardiology*.

eFigure 7. Kaplan-Meier Event-Free Survival Curves Stratified by RSF-Derived Risk Categories and Mayo Staging System

Kaplan-Meier event-free survival curves for the Bern-Swiss, Other-Swiss, and Vienna cohorts (upper, middle, and lower panels, respectively), stratified by RSF model-derived risk category (left panels) and Mayo staging system (right panels).

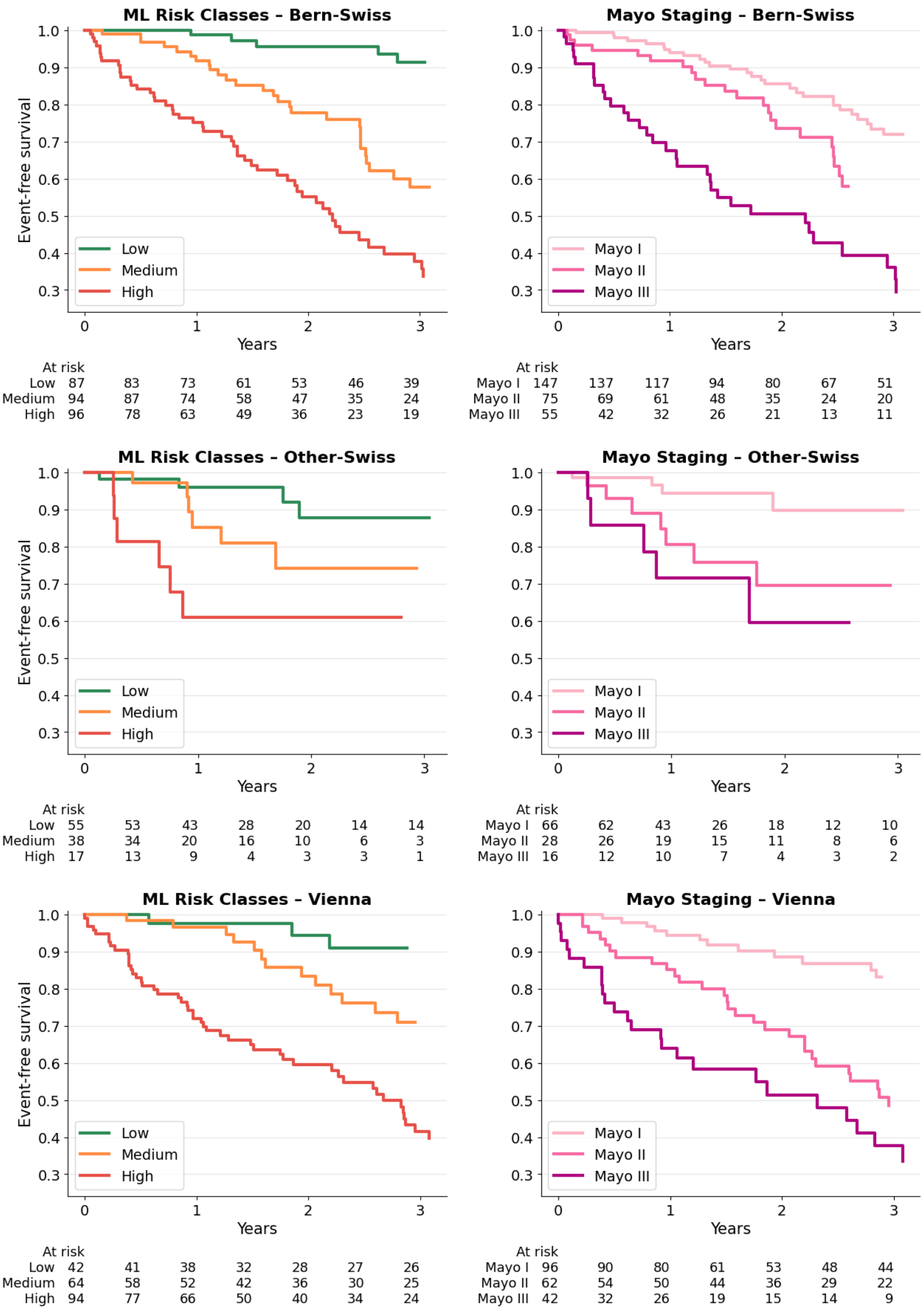

© 2026 Baj G et al. *JAMA Cardiology*.

eTable 1. Events Count and Follow-Up Duration

Number of patients and events by cohort, with median follow-up time. Values for the composite event (all-cause mortality and heart failure hospitalization) are reported as n (%); follow-up as median [IQR]. Abbreviations: HFH, heart failure hospitalization; FUP, follow-up.

	Patients	Events (%)	Death	HFH	Median FUP (months)
**Overall**	850	302 (35.5)	105	197	34 [15, 59]
**Bern-Swiss**	352	140 (39.8)	43	97	38 [20, 56]
**Swiss-Other**	260	53 (20.4)	12	41	20 [12, 38]
**Vienna**	238	109 (45.8)	50	59	53 [21, 82]

© 2026 Baj G et al. *JAMA Cardiology*.

eTable 2. Missing Values Count per Feature and Cohort

Overview of missing values across all features used in the analysis. For each feature, the absolute count and percentage of missing values are reported overall and by dataset split.

	Overall (850)	Bern-Swiss (352)	Other-Swiss (260)	Vienna (238)
**Mayo Score**	263 (30.9)	75 (21.3)	150 (57.7)	38 (16.0)
**Sodium**	128 (15.1)	32 (9.1)	71 (27.3)	25 (10.5)
**Maximum Wall Thickness**	124 (14.6)	56 (15.9)	50 (19.2)	18 (7.6)
**Hemoglobin**	119 (14.0)	25 (7.1)	94 (36.2)	0 (0.0)
**LVEDD**	119 (14.0)	43 (12.2)	57 (21.9)	19 (8.0)
**NAC Score**	102 (12.0)	39 (11.1)	62 (23.8)	1 (0.4)
**Potassium**	99 (11.6)	24 (6.8)	47 (18.1)	28 (11.8)
**NT-proBNP**	74 (8.7)	30 (8.5)	43 (16.5)	1 (0.4)
**eGFR**	60 (7.1)	21 (6.0)	39 (15.0)	0 (0.0)
**Mitral Valve Disease > Grade I**	50 (5.9)	10 (2.8)	23 (8.8)	17 (7.1)
**Aortic Valve Disease > Grade I**	49 (5.8)	11 (3.1)	22 (8.5)	16 (6.7)
**LVEF**	47 (5.5)	5 (1.4)	15 (5.8)	27 (11.3)
**Tricuspid Valve Disease > Grade I**	47 (5.5)	9 (2.6)	21 (8.1)	17 (7.1)
**Any Valve Disease > Grade I**	44 (5.2)	9 (2.6)	19 (7.3)	16 (6.7)
**Heart Rate**	32 (3.8)	13 (3.7)	4 (1.5)	15 (6.3)
**NYHA Class**	21 (2.5)	7 (2.0)	6 (2.3)	8 (3.4)
**BMI**	14 (1.6)	9 (2.6)	5 (1.9)	0 (0.0)
**Diabetes Mellitus**	11 (1.3)	5 (1.4)	6 (2.3)	0 (0.0)
**Pacemaker Implanted**	9 (1.1)	4 (1.1)	5 (1.9)	0 (0.0)
**History of Valvular Heart Disease**	8 (0.9)	2 (0.6)	6 (2.3)	0 (0.0)
**Arterial Hypertension**	5 (0.6)	0 (0.0)	5 (1.9)	0 (0.0)
**Sex**	1 (0.1)	0 (0.0)	1 (0.4)	0 (0.0)
**SGLT2 Inhibitor Therapy**	1 (0.1)	0 (0.0)	0 (0.0)	1 (0.4)
**Age at Baseline**	1 (0.1)	0 (0.0)	1 (0.4)	0 (0.0)
**MRA Therapy**	0 (0.0)	0 (0.0)	0 (0.0)	0 (0.0)
**History of Cancer**	0 (0.0)	0 (0.0)	0 (0.0)	0 (0.0)
**Stroke / TIA**	0 (0.0)	0 (0.0)	0 (0.0)	0 (0.0)
**Beta-blocker Therapy**	0 (0.0)	0 (0.0)	0 (0.0)	0 (0.0)
**Amyloid-specific Therapy**	0 (0.0)	0 (0.0)	0 (0.0)	0 (0.0)
**Oral Anticoagulant Therapy**	0 (0.0)	0 (0.0)	0 (0.0)	0 (0.0)
**RAAS Inhibitor Therapy**	0 (0.0)	0 (0.0)	0 (0.0)	0 (0.0)
**Diuretic Therapy**	0 (0.0)	0 (0.0)	0 (0.0)	0 (0.0)
**AF / AFL**	0 (0.0)	0 (0.0)	0 (0.0)	0 (0.0)

© 2026 Baj G et al. *JAMA Cardiology*.

eTable 3. Baseline Characteristics by Cohort for Patients With Available Staging Scores

Baseline characteristics of patients with available NAC and Mayo staging scores, by centre. Continuous variables are reported as median [IQR].

		Overall	Bern-Swiss	Other-Swiss	Vienna
**NAC**	**N**	748	313	198	237
	**Events**	270 (36.1%)	122 (39.0%)	39 (19.7%)	109 (46.0%)
	**NT-proBNP**	1757 [840–3344]	1697 [863–3201]	1620 [833–2996]	1929 [839–3785]
	**eGFR**	65 [50–78]	67 [51–81]	68 [56–80]	61 [45–75]
**Mayo**	**N**	587	277	110	200
	**Events**	208 (35.4%)	104 (37.5%)	19 (17.3%)	85 (42.5%)
	**NT-proBNP**	1734 [810–3212]	1655 [814–3142]	1554 [793–2862]	1917 [828–3494]
	**Troponin-T**	42 [30–62]	46 [31–62]	36 [26–56]	42 [29–64]

© 2026 Baj G et al. *JAMA Cardiology*.

eTable 4. Model Performance in the Treated Subgroup

Discrimination and calibration performance of the RSF model on the amyloid-specific treated cohort, evaluated by internal-external cross-validation. Values: estimate (95% CI).

		Bern-Swiss	Other-Swiss	Vienna
		**Discrimination**		
**C-index**	**Harrell**	0.67 (0.59–0.74)	0.74 (0.65–0.82)	0.72 (0.65–0.79)
	**Uno**	0.64 (0.56–0.72)	0.72 (0.63–0.81)	0.71 (0.63–0.79)
	**Antolini**	0.68 (0.62–0.75)	0.72 (0.62–0.82)	0.73 (0.66–0.80)
**AUC**	**1 Year**	0.78 (0.67–0.90)	0.75 (0.59–0.88)	0.74 (0.61–0.85)
	**2 Year**	0.68 (0.58–0.78)	0.72 (0.60–0.83)	0.75 (0.65–0.85)
	**3 Year**	0.67 (0.56–0.77)	0.82 (0.71–0.93)	0.75 (0.65–0.85)
		**Calibration**		
**Mean Calibration**	**1 Year**	0.50 (0.22–0.83)	0.98 (0.50–1.51)	0.74 (0.42–1.10)
	**2 Year**	1.03 (0.74–1.35)	0.97 (0.60–1.38)	0.78 (0.54–1.03)
	**3 Year**	1.02 (0.78–1.26)	0.77 (0.46–1.06)	0.88 (0.67–1.09)
**Slope**	**1 Year**	0.61 (0.29–1.00)	0.98 (0.60–1.40)	1.11 (0.74–1.65)
	**2 Year**	0.68 (0.33–1.12)	1.21 (0.72–1.78)	1.19 (0.78–1.80)
	**3 Year**	0.65 (0.29–1.10)	1.45 (0.93–2.09)	1.30 (0.81–1.97)
**ICI**	**1 Year**	0.06 (0.03–0.08)	0.02 (0.004–0.06)	0.003 (0.002–0.08)
	**2 Year**	0.03 (0.01–0.11)	0.01 (0.01–0.11)	0.04 (0.01–0.11)
	**3 Year**	0.04 (0.01–0.15)	0.05 (0.02–0.14)	0.08 (0.03–0.15)

© 2026 Baj G et al. *JAMA Cardiology*.

eTable 5. Discrimination Performance on Individual Endpoint Components by Cohort

Discrimination metrics are reported for HF hospitalisation (HFH) and all-cause mortality (Death) separately, evaluated on each held-out centre in an internal-external cross-validation framework. C-index values (Harrell, Uno, Antolini) and time-dependent AUC at 1, 2, and 3 years are shown as point estimates with 95% bootstrap confidence intervals. Abbreviations: N, number of patients; Events, number of observed sub-events in the held-out centre.

				C-index			AUC		
		**N**	**Events**	**Harrell**	**Uno**	**Antolini**	**1 Year**	**2 Year**	**3 Year**
**Bern-Swiss**	**HFH**	352	98	0.73 (0.67–0.78)	0.69 (0.63–0.74)	0.73 (0.67–0.78)	0.79 (0.71–0.86)	0.75 (0.68–0.82)	0.72 (0.65–0.80)
	**Death**	352	91	0.76 (0.71–0.81)	0.74 (0.67–0.81)	0.77 (0.72–0.82)	0.90 (0.84–0.95)	0.74 (0.66–0.83)	0.75 (0.67–0.83)
**Other-Swiss**	**HFH**	260	45	0.74 (0.66–0.82)	0.72 (0.64–0.80)	0.73 (0.65–0.81)	0.78 (0.66–0.88)	0.71 (0.60–0.81)	0.78 (0.68–0.88)
	**Death**	260	16	0.79 (0.67–0.89)	0.76 (0.62–0.89)	0.77 (0.65–0.88)	0.84 (0.65–0.99)	0.76 (0.61–0.89)	0.76 (0.59–0.91)
**Vienna**	**HFH**	238	61	0.69 (0.63–0.76)	0.65 (0.57–0.74)	0.70 (0.64–0.77)	0.80 (0.71–0.88)	0.69 (0.59–0.79)	0.70 (0.60–0.79)
	**Death**	238	86	0.78 (0.72–0.82)	0.79 (0.73–0.84)	0.78 (0.73–0.83)	0.79 (0.65–0.89)	0.78 (0.70–0.85)	0.82 (0.75–0.88)

© 2026 Baj G et al. *JAMA Cardiology*.

eTable 6. Cox Model Discrimination and Calibration by Cohort

Discrimination and calibration performance of the Cox model, evaluated by internal-external cross-validation. Results are shown for the whole and treated cohorts. Values: estimate (95% CI).

		Whole cohort			Treated cohort		
		**Bern-Swiss**	**Other-Swiss**	**Vienna**	**Bern-Swiss**	**Other-Swiss**	**Vienna**
				**Discrimination**			
**C-index**	**Harrell**	0.72 (0.67–0.76)	0.75 (0.67–0.82)	0.72 (0.67–0.76)	0.61 (0.53–0.69)	0.70 (0.58–0.80)	0.71 (0.64–0.78)
	**Uno**	0.70 (0.65–0.75)	0.74 (0.67–0.82)	0.71 (0.65–0.76)	0.61 (0.53–0.70)	0.70 (0.59–0.80)	0.72 (0.63–0.79)
	**Antolini**	0.72 (0.68–0.76)	0.75 (0.68–0.82)	0.72 (0.67–0.76)	0.61 (0.53–0.69)	0.70 (0.59–0.80)	0.71 (0.65–0.78)
**AUC**	**1 Year**	0.78 (0.70–0.85)	0.78 (0.68–0.87)	0.73 (0.64–0.82)	0.61 (0.45–0.77)	0.68 (0.53–0.83)	0.70 (0.57–0.82)
	**2 Year**	0.71 (0.64–0.78)	0.73 (0.63–0.82)	0.74 (0.66–0.81)	0.61 (0.50–0.72)	0.72 (0.60–0.82)	0.78 (0.69–0.87)
	**3 Year**	0.74 (0.68–0.80)	0.79 (0.70–0.88)	0.75 (0.68–0.83)	0.67 (0.56–0.77)	0.83 (0.72–0.93)	0.78 (0.67–0.87)
				**Calibration**			
**Mean Calibration**	**1 Year**	0.82 (0.60–1.05)	1.05 (0.70–1.41)	1.02 (0.75–1.31)	0.46 (0.20–0.75)	0.73 (0.36–1.15)	0.74 (0.38–1.09)
	**2 Year**	1.01 (0.82–1.22)	0.90 (0.64–1.17)	0.94 (0.76–1.17)	1.01 (0.74–1.31)	0.87 (0.55–1.23)	0.81 (0.55–1.09)
	**3 Year**	1.06 (0.92–1.22)	0.70 (0.49–0.90)	1.01 (0.85–1.19)	1.01 (0.78–1.28)	0.69 (0.44–0.96)	0.91 (0.69–1.13)
**Slope**	**1 Year**	1.18 (0.94–1.49)	2.25 (1.56–3.20)	1.12 (0.86–1.57)	0.83 (0.25–1.49)	2.63 (1.58–3.91)	1.52 (1.02–2.12)
	**2 Year**	1.18 (0.94–1.49)	2.25 (1.56–3.20)	1.12 (0.86–1.57)	0.83 (0.25–1.49)	2.63 (1.58–3.91)	1.52 (1.02–2.12)
	**3 Year**	1.18 (0.94–1.49)	2.25 (1.56–3.20)	1.12 (0.86–1.57)	0.83 (0.25–1.49)	2.63 (1.58–3.91)	1.52 (1.02–2.12)
**ICI**	**1 Year**	0.02 (0.01–0.05)	0.03 (0.02–0.06)	0.02 (0.01–0.07)	0.07 (0.04–0.09)	0.03 (0.02–0.07)	0.02 (0.01–0.08)
	**2 Year**	0.02 (0.01–0.07)	0.06 (0.04–0.11)	0.01 (0.01–0.09)	0.07 (0.01–0.09)	0.06 (0.04–0.12)	0.03 (0.01–0.11)
	**3 Year**	0.03 (0.01–0.07)	0.10 (0.07–0.16)	0.02 (0.01–0.10)	0.10 (0.01–0.12)	0.10 (0.06–0.17)	0.06 (0.02–0.14)

© 2026 Baj G et al. *JAMA Cardiology*.
