# Supplementary material for: Machine Learning–Driven Risk Prediction Model in Transthyretin Amyloid Cardiomyopathy
Source: JAMA Cardiol. 2026 Aug 28;11(10):932–41. doi: 10.1001/jamacardio.2026.3496 (PMC13525483; doi:10.1001/jamacardio.2026.3496)
Supplement: Supplementary file 2 [file jamacardiol-e263496-s002.pdf]

Data Sharing Statement

Baj. Machine Learning–Driven Risk Prediction Model in Transthyretin Amyloid Cardiomyopathy. *JAMA Cardiol*. Published August 28, 2026.
doi:10.1001/jamacardio.2026.3496

Data
Data available: Yes
Data types: Participant data with identifiers
How to access data: The fully anonymized datasets from Bern and Vienna are available for the authors only under restricted access in accordance with institutional review board (IRB) approvals and applicable data-sharing regulations. Due to data privacy laws, the raw datasets cannot be made publicly available. Access may be granted upon approval by the IRBs and Data Sharing Committees of Bern and Vienna and the requesting institution. Approved requests will be processed within a one-year timeframe. Details on how to request access are available from the corresponding author upon reasonable request.
When available: With publication

Supporting Documents
Document types: Statistical/analytic code
How to access documents: Model development and hyperparameter tuning were performed with scikit-learn and scikit-survival packages in Python 3.10. Calibration curves and statistical tests were performed with R 4.2. All developed code and models are publicly available on our AI-CVM Laboratory GitHub repository (https://github.com/AI-in-Cardiovascular-Medicine/AiTTRIX).). Moreover, we have provided a publicly available web service, AiTTRIX (Artificial Intelligence for TransThyretin cardiac amyloidosis RIsk prediction using an eXplainable model), accessible via the following link (https://ai-cvm.com/software/AiTTRIX), that allows users to easily upload their data to obtain a risk score based on models developed in the current study.
When available: With publication

Additional Information
Who can access the data: researchers whose proposed use of the data has been approved
Types of analyses: For Research only.
Mechanisms of data availability: Access may be granted upon approval by the IRBs and Data Sharing Committees of Bern and Vienna and the requesting institution. Approved requests will be processed within a one-year timeframe. Details on how to request access are available from the corresponding author upon reasonable request.
